# Women who develop ovarian cancer show an increase in serum calcium and a decrease in serum albumin. A longitudinal study in the Janus Serum Bank Cohort

**DOI:** 10.1016/j.ygyno.2020.07.006

**Published:** 2020-07-25

**Authors:** Gary G. Schwartz, Steinar Tretli, Marilyn G. Klug, Trude E. Robsahm

**Affiliations:** a Department of Population Health, University of North Dakota School of Medicine & Health Sciences, Grand Forks, ND, USA; b Department of Research, Cancer Registry of Norway, Oslo, Norway

**Keywords:** Ovarian cancer, Calcium, Albumin, Biomarkers, Cancer screening

## Abstract

**Background.:**

Ovarian cancer is associated with high serum calcium and low serum albumin in clinical and epidemiologic studies. Whether high calcium and low albumin predispose to ovarian cancer or reflect existing cancer is unclear.

**Objective.:**

Test the hypothesis that serum calcium increases and serum albumin decreases in women who develop ovarian cancer.

**Methods.:**

Two hundred and four women donated sera to the Janus Serum Bank in Norway pre- and post-diagnosis of ovarian cancer, donations separated by approximately 14 years. We measured calcium and albumin in these sera and calculated the albumin-corrected calcium. Sera were adjusted for patient age and storage time.

**Results.:**

Post-diagnosis, mean age- and storage-adjusted calcium increased, from 2.53 to 2.68 mmol/L (*p* < .001). Mean age- and storage-adjusted, albumin-corrected calcium increased from 2.3 to 2.7 mmol/L (*p* < .001). Conversely, mean age- and storage-adjusted albumin decreased, from a mean of 51.3 to 40.9 g/L (*p* < .001). Significant changes were observed in women with early stage and metastatic cancer.

**Conclusions.:**

These data support the hypothesis that calcium and albumin are serum biomarkers of extant ovarian cancer. Longitudinal changes in calcium and albumin may be useful in ovarian cancer early detection.

## 1. Introduction

In the U.S., ovarian cancer is the 5th leading cause of cancer mortality and accounts for 5% of cancer deaths among women. Survival from ovarian cancer is highly stage-dependent. Most ovarian tumors are detected at Stages III and IV, for which the 5-year survival rate is 30%. Conversely, if ovarian cancer is detected at Stage I, before it spreads beyond the ovary, the 5-year survival is 92% [[1]]. However, only 15% of ovarian cancers are detected at this early stage. Thus, biomarkers that identify ovarian cancers at early stages are urgently needed.

Data from population-based studies suggest that serum calcium and albumin could serve as ovarian cancer biomarkers. For example, we examined stored sera from participants in the Janus Serum Bank, a population-based cohort in Norway that enrolled women without clinical cancer [[2]]. In a nested case-control study comparing cases who subsequently developed ovarian cancer to controls who did not, cases had significantly higher serum calcium 2–15 years prior to diagnosis [[3]]. Additionally, in a series of 514 U.S. women who underwent surgery for an adnexal mass that was suspicious for malignancy, women with ovarian masses that were malignant, including women with early stage cancers, had significantly higher serum calcium and significantly lower serum albumin than women with ovarian masses that were benign [[4]]. These findings are consistent with the hypothesis, originally proposed by Schwartz and Skinner, that the processes of ovarian carcinogenesis cause serum calcium levels to rise and serum albumin levels to fall [[5]]. The rise in serum calcium is believed to result from the upregulation of factors like parathyroid hormone-related protein [PTHrP], which release calcium from bone into blood [[6]]. The fall in albumin likely has several causes, including the inhibition albumin synthesis, sequestration of albumin in ascites or pleural effusions, and bowel obstruction [[7] [8]].

The finding that levels of serum calcium and albumin discriminate malignant from benign ovarian masses in the surgical setting suggests that these analytes might identify ovarian cancers in a screening setting. However, the studies published to date include data on calcium and albumin from a single time point only. Thus, they cannot differentiate between the hypothesis that high calcium and low albumin are markers of existing cancer (henceforth, “*the carcinogenesis hypothesis*”), from the alternative hypothesis that the levels of these analytes are associated with a higher lifetime risk of ovarian cancer but do not reflect existing cancer (“*the genetic hypothesis*”). If the carcinogenesis hypothesis is correct, calcium and albumin could be useful in cancer screening; if the genetic hypothesis is correct, they would not be (although their levels might define a population at increased risk). Discriminating between these hypotheses is important and requires blood collected at at least two time points: at the time of cancer diagnosis and at a time significantly prior to it. That is, compared to their levels pre-diagnosis, the carcinogenesis hypothesis predicts that, post-diagnosis, serum calcium should increase and serum albumin should decrease. Conversely, the genetic hypothesis predicts that the levels of these analytes post-diagnosis should not differ from their levels pre-diagnosis.

A unique feature of the Janus Serum Bank is that, in addition to the serum sample banked from women prior to their ovarian cancer diagnosis, it contains a second sample from these women post- diagnosis, prior to the initiation of therapy. Thus, by comparing the calcium and albumin levels pre- and post-diagnosis, the Janus cohort offers an opportunity to differentiate between the carcinogenesis and genetic hypotheses.

## 2. Methods

### 2.1. Janus Serum Bank Cohort

The Janus Serum Bank Cohort is a population-based biobank established in 1973, a detailed description of which is published else-where [[9] [10]]. Briefly, Janus includes more than 330,000 serum samples from approximately 317,000 adult donors. Ninety one percent (91%) of the participants are from population-based health surveys and 9% are from blood donors in Oslo. Participants were residents of 17 of Norway’s 19 counties and were aged 35–49 years at the time of their first blood donation.

Cancer diagnoses in Norway since 1953 have been recorded by the Cancer Registry of Norway (CRN). The completeness of registration is estimated at >98% [[11]]. Record linkage between the CRN and Janus identified Janus participants with histologically-verified diagnoses of invasive ovarian cancer (“cases”). Eligible cases were women who donated serum at least 2 years prior to their cancer diagnosis. Women who subsequently developed ovarian cancer and were treated at the Norwegian Radium Hospital donated a second serum sample, prior to cancer treatment. All serum samples have been stored at −25 °C.

Personal data were de-identified before statistical analyses. Approvals for this study were obtained from the Janus Serum Bank Board and the Regional Committees for Medical and Health Research Ethics in Norway and from the IRB of the School of Medicine & Health Sciences at the University of North Dakota (IRB Number 202006–309).

### 2.2. Serum measurements

For each participant, 70 μL serum was extracted for analysis of total serum calcium and albumin. Calcium and albumin were measured by an automatic analyzer (Roche Modular P), as described [11]. Albumin was measured using bromocresol green. Participating laboratories were accredited by the Norwegian Standards for Testing and Calibration (NS-ISO/IEC 17025). Validation studies of stored serum samples demonstrate high stability of calcium [[12]] and albumin [[13]]. The coefficient of variation (cv) for total serum calcium was 2.0% at 2.44 and 3.27 mmol/L and was <2.4% at 37.1 and 40.3 g/L for serum albumin.

### 2.3. Statistical adjustment for age and storage time

Serum calcium and albumin levels are influenced by a woman’s age. Similarly, time in storage can affect the concentration of analytes in serum. Thus, in order to make valid inferences about the effect of ovarian cancer on serum calcium and albumin, the effects of cancer *per se* must be separated from the effects of aging and storage time.

### 2.4. Calcium, adjustment for age

We used calcium values from women at seven ages across the lifespan and derived a cubic equation predicting expected calcium levels from age. Two equations were used to adjust for age: one for women under 70 and one for women 70 and older, as there was a significant shift in parameters at that age. For younger women, calcium generally increased with time. The equation describing this was: CA = 2.642−0.02130*Age + 0.0004830*Age^*2*^−0.000003269*Age^*3*^. For women seventy or older, calcium levels increased slightly. The resultant equation for these women was: *CA* = 2.410−0.006104*Age + 0.0001671*Age^*2*^−0.000001170*Age [3].

### 2.5. Calcium, adjustment for storage time

Calcium levels in the Janus cohort have been reported to increase with time in storage (Gislefoss, et al., 2008). Logarithmic equations were used to account for no increase immediately after storage and a 6.4% increase after two years. To adjust serum calcium for the effects of storage, we used the equation: *% Increase* = 0.4 * ln(*Years −0.0000002201*) *+ 6.137*.

### 2.6. Albumin, adjustment for age

In published data on albumin and age for Norwegian women, age was not a significant predictor of albumin levels. The Norwegian Oslo Health Study reported that, among 1692 stroke-free Oslovian women aged <45 years, the mean albumin was 46.6 g/L (± 0.1). For stroke-free Oslovian women >59 years, the mean was 45.7 g/L (± 0.1) [[14]]. For our study, we subtracted 0.9 g/L from the albumin at the second serum donation if the woman’s age was <45; otherwise, no adjustment was applied.

### 2.7. Albumin, adjustment for storage time

Gislefoss et al. compared serum albumin values in 130 samples from the Janus Serum Bank after 25 years in storage. The albumin values increased very modestly, from 44.5 (42.8–45.8) to 45.0 g/L (43.3–47.1). To adjust albumin for storage time, 0.28 X (length of storage in years) was subtracted from the albumin.

### 2.8. Albumin-corrected serum calcium

An accurate estimate of the calcium that is biologically active depends upon the concentration of serum albumin, as only the unbound (“free” or “ionized”) fraction of serum calcium is active. Each 1 g/dL of albumin binds 0.2 mmol (0. 8 mg/dL) of calcium [[15]]. Thus, for every gram of albumin below or above 4.0, the serum calcium is corrected, up or down (respectively), by 0.2 mmol. Adjustment was made for both hypo- and hyper-albuminemia. The age- and storage-adjusted values were then used in a standard equation, *Albumin-corrected calcium* = Calcium + 0.02 * (*40 − Albumin*) [[16]].

### 2.9. Comparison of calcium and albumin to expected values

In Janus, only individuals with cancer donated a second serum sample. Thus, in order to compare changes in serum calcium and albumin among cases at the time of diagnosis to changes that would be expected among women without ovarian cancer, we used data from our previous case-control study for the controls in this cohort [3]. We used the mean serum albumin of controls (50.7 g/L) and estimated standard deviation (3.0 g/L) for the pre-diagnosis value. The post-diagnosis albumin value was derived by adjusting for age, as described. Ninety-five percent (95%) confidence intervals were estimated pre- and post-diagnoses. The average calcium for non-cancer controls was 2.5 mmol/L, and the estimated SD was 0.183 mmol/L. This value was age-adjusted for the average pre-diagnosis age of 42.7 years and the post-diagnosis age of 57.5 years, using the adjustment formula: *2.642 −0.0213 Age + 0.000483 Age*^*2*^ *− 0.000003269 Age*^*3*^ *and 2.410−0.006104 Age + 0.0001671 Age*^*2*^ *− 0.00000117 Age*^*3*^ (*pre-diagnosis and post-diagnosis, respectively*).

Paired *t*-tests were used to compare serum values between Times 1 and 2 for all observations and within individual stages. Differences in values between Times 1 and 2 were compared between stages using general linear models with Tukey’s multiple comparison tests.

## 3. Results

Background data on this study population were reported previously [3]. Briefly, we identified 208 cases in JANUS with matching sera before and after diagnosis of ovarian cancer during the time period 1978–2004. After exclusions due to missing values (*N* = 4), data remained for 204 participants (98%).

Characteristics of women participating in JANUS are shown in Table 1. The average age of women who subsequently developed ovarian cancer at entry into the cohort was 42.7 years (SD = 3.7) and at diagnosis, was 57.5 (SD = 5.8). On average, women were 165.35 cm (147–185) tall and their average body mass index (kg/m^2^) was 24.23 (15.4–40.8). Thirty-eight (38) women (19%) had localized disease at diagnosis, 155 had metastatic disease (76%) and the stage of 11 (6%) was unknown.

Data on the unadjusted and time- and storage-adjusted values for calcium, albumin, and albumin-corrected calcium also are shown in Table 1. After the diagnosis of ovarian cancer, age- and storage-adjusted serum calcium levels increased significantly, from 2.53 to 2.68 mmol/L (*p* < .001). Values for age- and storage-adjusted, albumin-corrected serum calcium increased by ~15% (*p* > .001), from 2.3 to 2.7 mg/dL. Serum albumin levels, corrected for age and storage-time, declined by >20%, from a mean of 51.3 to 40.94 g/L (*p* < .001).

Age- and storage-adjusted serum calcium increased significantly both in women with localized disease and in women with metastatic disease. Similarly, age- and storage-adjusted serum albumin declined significantly in women with localized disease and in women with metastatic disease. The decline in serum albumin among women with metastatic cancer was significantly greater than the decline among women with localized disease (22% vs. 12.7%; *p* > .01).

**Fig. 1.**
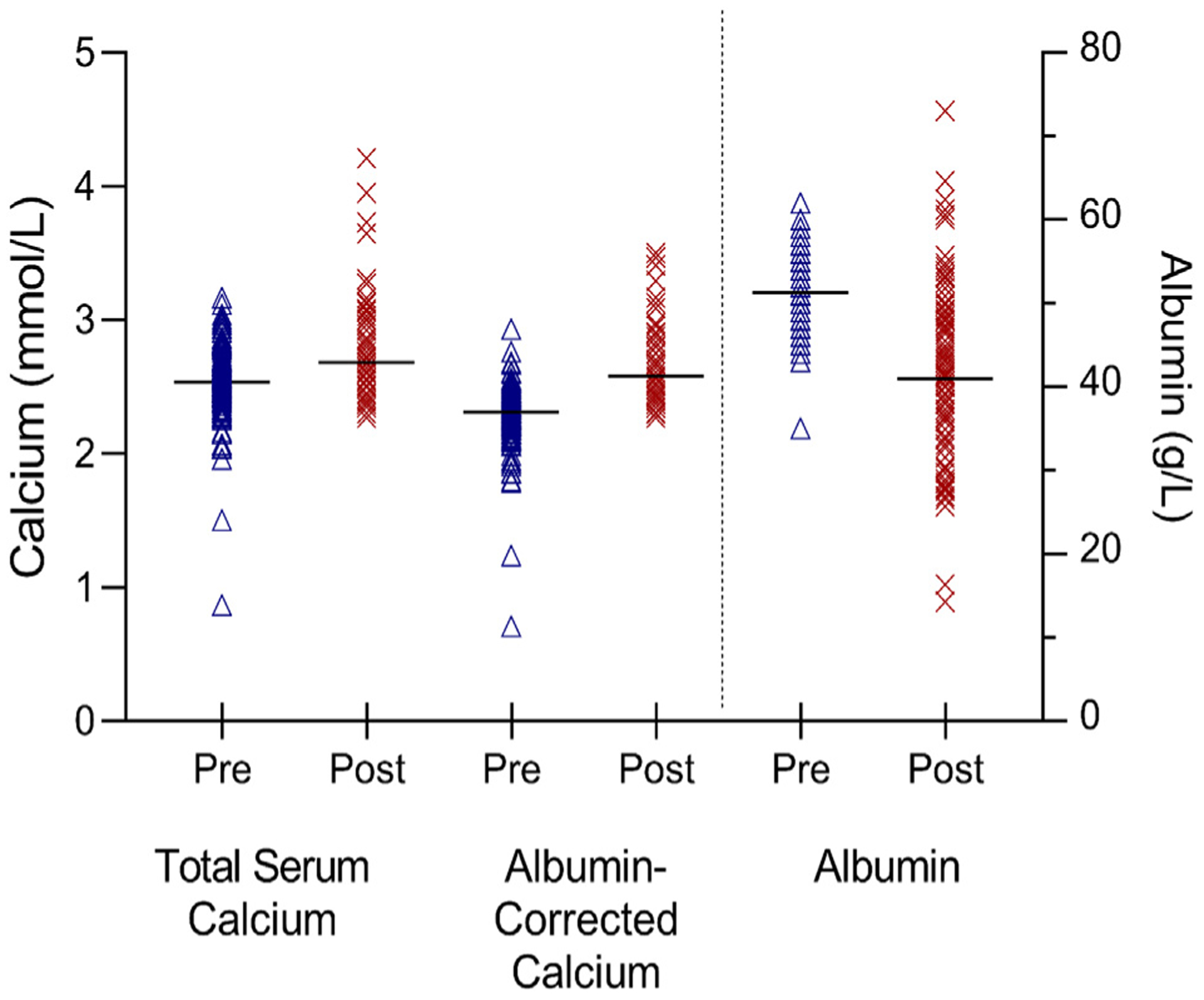
Total serum calcium, albumin-corrected serum calcium, and albumin levels in women in the Janus Serum Bank Cohort pre- and post-diagnosis of ovarian cancer. Mean values are shown by horizontal bars. Samples are separated in time by an average of 14 years and are adjusted for womens’ ages and the time that the samples have been in frozen storage (as described in Methods, 2.3–2.8).

**Fig. 2.**
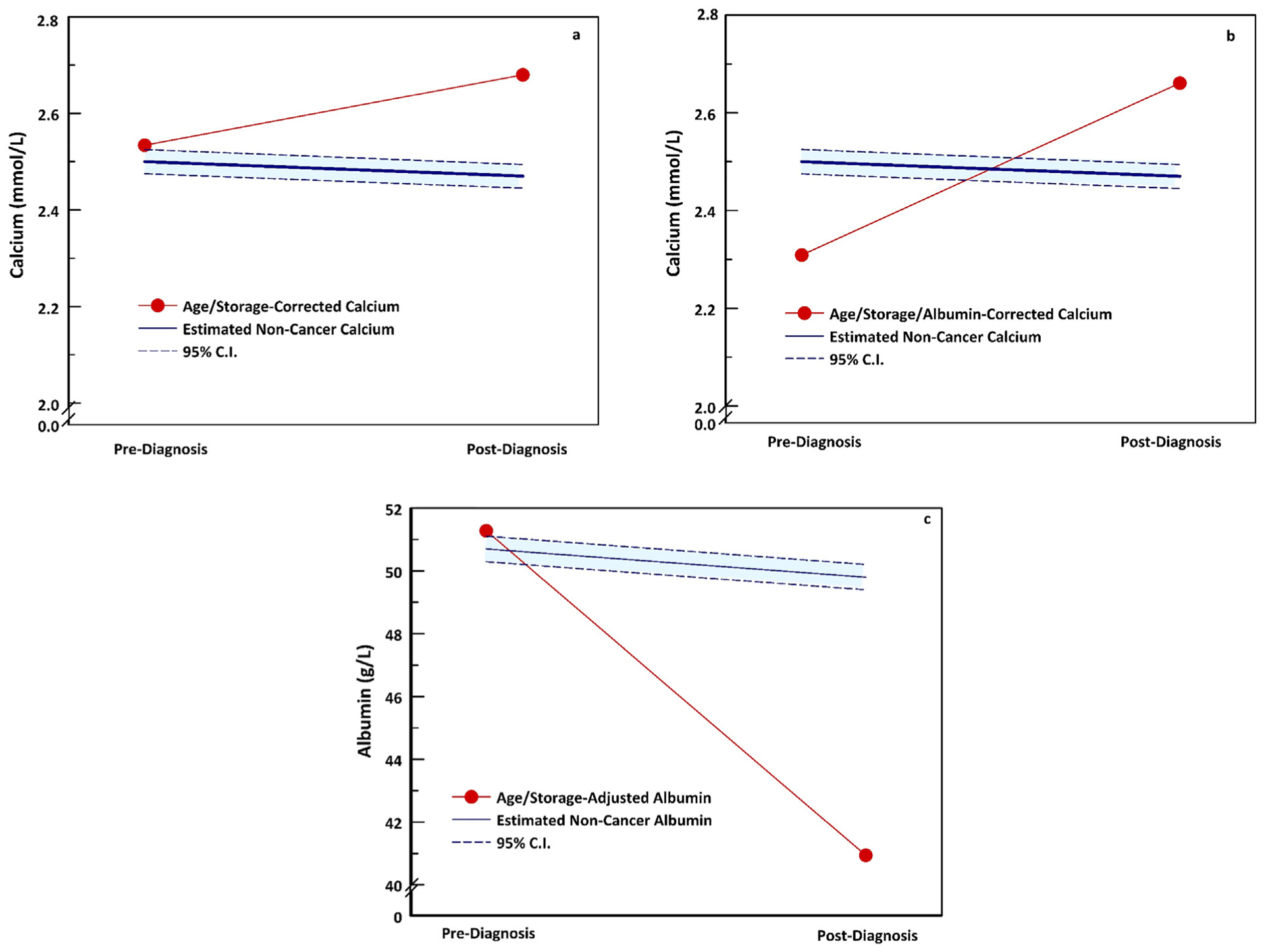
a,b,c. Changes in age- and storage-adjusted serum calcium (a), age- and storage- albumin-corrected serum calcium (b), and age- and storage corrected serum albumin (c) in women before and after the diagnosis of ovarian cancer (red lines). Dotted blue lines show the 95% Confidence Interval for projected changes in the absence of ovarian cancer derived from our case-control study. The controls were age-matched Norwegian women without a subsequent diagnosis of ovarian cancer from the Janus Serum Bank Cohort, as described in Methods, 2.9.

Changes in total serum calcium, albumin-corrected calcium, and serum albumin are shown in Fig. 1, which compares these values pre- and post-diagnosis (i.e., Time 1 and Time 2). Fig. 2 shows the relationship of the actual values among cases (red lines) to actual values observed among control women without cancer at Time 1 and the projected values after diagnosis among the cases at Time 2 (blue lines).

## 4. Discussion

We analyzed sera from women from a population-based cohort in Norway who donated blood at two time points: Time 1, prior to the diagnosis of ovarian cancer, and Time 2, post-ovarian cancer diagnosis, approximately 14 years later. Our most important conclusions are that after adjustment for a woman’s age and for the time that samples were frozen, serum calcium levels increased significantly and serum albumin levels decreased significantly. Importantly, significant changes in age- and storage-adjusted calcium and albumin were observed in women with localized as well as metastatic cancer. These data support the hypothesis that the increases in calcium and decreases in albumin reflect the effects of extant cancer.

After adjustment for age and storage time, total calcium levels increased post-diagnosis from 2.53 to 2.68 mmol/L (a 6% increase; *p* < .001). This increase is notable because the concentration of ionized calcium, the biologically active fraction of total serum calcium, is tightly regulated by parathyroid hormone (PTH) and normally does not deviate from its set point by more than 2% [[17]]. The stability of ionized calcium contributes to the stability of total serum calcium, of which 50% is ionized. Higher ionized calcium levels recently were reported in association with lymph node metastases among women with endometrial cancer, a malignancy that shares pathophysiologic features with ovarian cancer [[18] [19]].

Compared to total serum calcium, albumin-corrected serum calcium is a more accurate measure of an individual’s calcium status. The combination of the post-diagnosis increase in total serum calcium and substantial decline in serum albumin resulted in a large increase in albumin-corrected serum calcium, from 2.31 to 2.66 mmol/L (~ 15%, *p* > .001). These data suggest that the true prevalence of hypercalcemia in ovarian cancer, i.e., of an albumin-corrected serum calcium >2.6 mmol/L, likely is higher than the estimate of 5% reported in the literature (which was based on uncorrected serum calcium) [[20]].

Instances of elevated serum calcium in ovarian cancer most often are a paraneoplastic effect resulting from the actions of PTHrP, the principal agent of hypercalcemia of malignancy [[21]]. PTHrP is an oncofetal protein secreted by many malignant cells, including ovarian cancer cells. PTHrP is structurally similar to PTH and binds to the PTH-type 1 receptor in bone, causing the release of calcium into circulation and inhibiting calcium excretion by the kidney [[22]]. Immunohistochemical studies demonstrate selective staining of PTHrP in malignant but not benign ovarian tumors [[23]].

We observed a large decline in serum albumin post-diagnosis of ovarian cancer (~ 20%). This was observed in both early stage and metastatic disease, with a larger decline among women with metastatic disease (*p* < .01). The association of ovarian cancer with hypoalbuminemia is well-known [[24]]. Ovarian cancers cause serum albumin to decline by several mechanisms, including bowel obstruction, loss of albumin to ascites, poor nutrition, and inhibition of albumin synthesis [[25]]. In early stage ovarian cancer, the fall in serum albumin likely results from metabolic factors secreted by ovarian cells, including interleukin-6 (IL-6), an inflammatory cytokine that inhibits the synthesis of albumin by hepatocytes and promotes the metastasis of ovarian cancer cells [[26]] [[27]].

It is important to consider possible alternative explanations for the effects we observed. Albumin levels are relatively stable in healthy women but can decline in several conditions, including malnutrition and malabsorption [[28]]. Serum calcium values also are very stable but can be increased by factors including thiazide diuretics, lithium and excess vitamins A and D [[29]]. In non-hospitalized individuals, the commonest cause of elevated serum calcium is primary hyperparathyroidism. It is conceivable that some women could have developed primary hyperparathyroidism in the interval from T1 to T2, causing their serum calcium to rise. However, the co-occurrence of ovarian cancer and primary hyperparathyroidism is considered exceptionally rare [[30]]. Moreover (to our knowledge), primary hyperparathyroidism does not cause serum albumin levels to decline [[31] [32]]. Thus, we conclude that primary hyperparathyroidism is unlikely to have had an important influence on these data.

Our study has several limitations, the most important of which is the absence of data for a comparison group of women without ovarian cancer at the time the cases were diagnosed. Because a second serum sample was obtained only from participants who developed cancer, we were unable to compare the changes in calcium and albumin among cases to those of women without cancer. However, the natural history of serum calcium and albumin is exceptionally well-studied in population-based cohorts, including the Janus and the Norwegian Oslo Health Study cohorts, which provided clear predictions for expected values among women without cancer (Fig. 2). In the future, this limitation could be overcome via studies with longitudinal serum samples from cases and controls. The relatively small number of women with localized disease (*N* = 38) is a limitation but is consistent with the stage distribution of ovarian cancers at diagnosis. However, it is noteworthy that in the women with early stage disease, serum levels of age- and storage- adjusted calcium and age- and storage-adjusted albumin showed significant changes post-diagnosis, similar to those of women with metastatic disease. This supports the view that changes in serum calcium and albumin may be useful in the early detection of ovarian cancer.

Conversely, our study has several strengths. First, the study is population-based, and the most important threat to its validity, loss to follow-up, is virtually zero. Secondly, the differences we observed in serum albumin and albumin-corrected serum calcium may be conservative. For example, Høstmark et al. reported an increase in serum albumin of 0.28 g/L per year in Janus samples after 22 years in frozen storage [[33]]. This was not confirmed by Gislefoss et al. (2008), who also used sera from Janus and reported little change in serum albumin with long-term storage. Our calculations used the estimates of Gislefoss et al. If albumin levels in healthy women in fact increased with storage time, the substantial decrease we observed post-diagnosis (20%) would be greater. Secondly, serum albumin was measured by bromocresol green, the method used in most laboratories [[34]]. Compared to the gold standard for albumin measurement, immunoassays, bromocresol green overestimates albumin in patients with inflammatory conditions due to its non-specific binding to serum globulins [[35] [36]]. Duly et al. showed that bromocresol green overestimated albumin in serum samples by ~5 g/L, with a greater effect on samples with low albumin [[37]]. This suggests that, relative to their values pre-diagnosis, the values for albumin we observed post-diagnosis may be overestimated (i.e., their true value may be lower). Similarly, the increase in albumin-corrected serum calcium may be underestimated (i.e., its true value may be higher).

In conclusion, our study of serum calcium and albumin in women pre- and post-diagnosis of ovarian cancer demonstrates that serum calcium levels increased significantly and serum albumin levels decreased significantly. These findings, which require confirmation in other prospective cohorts, are consistent with the predictions of the carcinogenesis hypothesis. If confirmed, they suggest that longitudinal changes in serum calcium and albumin could play a role in ovarian cancer screening/early detection. For example, calcium and albumin are commonly measured in a panel of analytes obtained on every medical visit, the Comprehensive Metabolic Panel (CMP). The CMP could automatically calculate changes in serum calcium and albumin as part of a woman’s annual physical exam. A pattern of rising serum calcium and falling serum albumin may be suspicious for ovarian cancer and could be used to refer women for further diagnostic testing, e.g., transvaginal ultrasound.

We emphasize that this is a pilot study and that it does not validate the use of serum calcium and albumin as part of a screening strategy for ovarian cancer. However, serum calcium and albumin are well-characterized analytes that are measured commonly, quickly, and inexpensively. Their potential role in screening for ovarian cancer should be tested in other established cohorts with sera banked prior to the clinical detection of ovarian cancer, such as the Prostate, Lung, Colorectal and Ovarian (PLCO) screening trial [[38]]. Future longitudinal studies, including those in which changes in calcium and albumin are added to panels of other biomarkers for ovarian cancer, are warranted [[39]].

## Figures and Tables

**Table 1:** Characteristics of participants.

	All			Local			Metastatic			Unknown		
	N	Mean	Std	N	Mean	Std	N	Mean	Std	N	Mean	Std
Age Time 1	204	42.735	3.708	38	43.763	4.553	155	42.445	3.466	11	43.273	3.438
Age Time 2	204	57.475	5.752	38	57.895	6.762	155	57.258	5.414	11	59.091	6.848
Years to Time 1	204	28.566	3.347	38	28.667	3.432	155	28.490	3.311	11	29.280	3.790
Years to Time 2	204	13.864	4.096	38	14.594	4.904	155	13.706	3.982	11	13.561	2.236
Unadjusted Albumin Time 1	204	51.279	3.461	38	51.579	3.438	155	51.245	3.280	11	50.727	5.746
Unadjusted Albumin Time 2	204	45.181	7.454	38	49.053	7.980	155	44.310	7.709	11	44.091	6.441
Comparisons	D = 6.098			D = 2.526			D = 6.936			D = 6.636		
	*t* = 11.33; *p* < .001			*t* = 1.74; *p* = .090			*t* = 12.05; *p* < .001			*t* = 3.00; *p* = .013		
Age/Storage Time 1 Adjusted Albumin	204	51.279	3.461	38	51.579	3.438	155	51.245	3.280	11	50.727	5.746
Age/Storage Time 2 Adjusted Albumin	204	40.937	8.060	38	45.041	8.696	155	40.031	7.709	11	39.526	6.684
Comparisons	D = 10.342			D = 6.538			D = 11.214			D = 11.201		
	*t* = 17.87; *p* < .001			*t* = 4.17; *p* < .001			*t* = 18.10; *p* < .001			*t* = 4.85; *p* < .001		
Unadjusted Calcium Time 1	204	2.534	0.237	38	2.548	0.264	155	2.532	0.227	11	2.529	0.298
Unadjusted Calcium Time 2	204	2.521	0.272	38	2.642	0.378	155	2.498	0.239	11	2.423	0.154
Comparisons	D = 0.013			D = 0.096			D = 0.033			D = 0.106		
	*t* = 0.56; *p* = .3577			*t* = 1.25; *p* = .219			*t* = 1.35; *p* = .178			*t* = 1.15; *p* = .276		
Age/Storage Adjusted Calcium Time 1	204	2.534	0.237	38	2.548	0.264	155	2.532	0.277	11	2.529	0.298
Age/Storage Adjusted Calcium Time 1	204	2.680	0.278	38	2.805	0.381	155	2.656	0.245	11	2.581	0.161
Comparisons	D = 0.146			D = 0.257			D = 0.124			D = 0.052		
	*t* = 6.18; *p* < .001			*t* = 3.47; *p* = .001			*t* = 5.19; *p* < .001			*t* = 0.59; *p* = .565		
Age/Storage Time 1/ Adjusted Albumin / Age/Storage Adjusted Calcium	204	2.309	0.209	38	2.316	0.257	155	2.307	0.196	11	2.315	0.215
Age/Storage Time 2/ Adjusted Albumin / Age/Storage Adjusted Calcium	204	2.661	0.193	38	2.704	0.230	155	2.656	0.186	11	2.590	0.118
Comparisons	D = 0.352			D = 0.388			D = 0.349			D = 0.275		
	*t* = 19.87; *p* < .001			*t* = 7.80; *p* < .001			*t* = 17.84; *p* < .001			*t* = 5.33; *p* < .001		

Time 1 to Time 2 differences in unadjusted albumin differed significantly between localized and metastatic cancer (F = 5.26, *p* = .006). The magnitude of the difference was significantly higher in metastatic than in localized disease (6.936 and 2.526) (Tukey’s *p* < .05).

Adjusted albumin levels differed significantly between stages (F = 5.15, *p* = .007). The difference between Time 1 and Time 2 was significantly higher in metastatic disease than in localized disease (11.214 and 6.538) (Tukey’s *p* < .05).

Time 1 to Time 2 differences in unadjusted calcium did not differ significantly between metastatic and local disease (F = 2.57, *p* = .079).

Time 1 to Time 2 differences in adjusted calcium did not differ significantly between metastatic and local disease (F = 0.89, *p* = .412).

## HIGHLIGHTS

- We measured frozen sera from 204 women pre- and post- diagnosis of ovarian cancer, approximately 14 years apart.
- Women who developed ovarian cancer showed significant increases in calcium and significant decreases in albumin.
- Longitudinal changes in serum calcium and serum albumin may be useful as biomarkers of ovarian cancer.
